# Injection of Viscous Micro-Droplet via Nozzle-Driven Piezoelectric Micro-Jet and Its Performance Control Method

**DOI:** 10.3390/mi14061267

**Published:** 2023-06-18

**Authors:** Ti-Yuan Shan, Xiao-Sheng Wu, Yuan-Wen Hu, Xin-Di Lin, Dan-Feng Sun

**Affiliations:** 1 School of Electronic Information and Electrical Engineering, Shanghai Jiao Tong University, Shanghai 200000, China; shantiyuan@sjtu.edu.cn; 2 Shanghai Aerospace Control Technology Institute, Shanghai 200000, China; huyuanwen@803.sast.casc (Y.-W.H.); linxindi@803.sast.casc (X.-D.L.); sundanfeng@803.sast.casc (D.-F.S.)

**Keywords:** piezoelectric micro-jet, performance control method, coupling analysis

## Abstract

The inkjet printing technology based on piezoelectric micro-jets can effectively realize the efficient and high-precision processing of special-shaped structures. In this work, a nozzle-driven piezoelectric micro-jet device is proposed, and its structure and micro-jet process are described. ANSYS two-phase, two-way fluid–structure coupling simulation analysis is carried out, and the mechanism of the piezoelectric micro-jet is described in detail. The effects of voltage amplitude, input signal frequency, nozzle diameter and oil viscosity on the injection performance of the proposed device are studied, and a set of effective control methods is summarized. The correctness of the piezoelectric micro-jet mechanism and the feasibility of the proposed nozzle-driven piezoelectric micro-jet device are proved by experiments, and an injection performance test is carried out. The experimental results are consistent with the ANSYS simulation results, which confirms the correctness of the experiment. Finally, the stability and superiority of the proposed device are verified via comparation experiments.

## 1. Introduction

Piezoelectric materials are widely used, and piezoelectric micro-jets are one of their important applications [1,2,3,4,5,6]. A piezoelectric micro-jet is a technology based on piezoelectric drive. When a pulse voltage is applied to a piezoelectric vibrator, the piezoelectric vibrator vibrates and generates acoustic pressure waves in the cavity. After the propagation and reflection of the pressure waves, the pressure waves propagating to the nozzle are superimposed on each other to make the droplet eject. Piezoelectric micro-jet technology can realize droplet injection on demand and has the advantages of high precision, fast response speed and high efficiency [7]. Due to its unique advantages, piezoelectric micro-jets have been widely used in many industrial fields, such as medical treatment [8], biology [9], underwater drive [10], micro-fluid filtration [11], additive manufacturing [12], etc.

Modern micro-structure products are developing rapidly in the direction of being compact, complicated and multi-functional, which requires a higher level of manufacturing and processing of products. Additive manufacturing technology was born under such a background [13]. Additive manufacturing technology, also commonly known as 3D printing, is a key enabling technology related to many industrial fields. It breaks through the limitations of traditional processing technology and realizes the efficient and high-precision processing of special-shaped structures [14,15]. At present, the additive manufacturing technologies used in the engineering field mainly include fused deposition modeling [16], selective laser sintering [17] and inkjet printing [18]. Among these technologies, the inkjet printing technology based on piezoelectric micro-jets has been paid more and more attention by researchers due to its advantages such as high printing accuracy, fast dynamic response, simple structure, no electromagnetic interference and green environment protection [19].

The existing piezoelectric micro-jet devices can be roughly divided into four structural types: squeeze type, bend type, push type and shear type [13]. Ulmke et al. [20] proposed a squeeze-type piezoelectric micro-jet device, which could generate droplets with a diameter ranging from 10 microns to 100 microns, which was equivalent to the volume of the ejected droplets as low as 0.6 pl. Li et al. [21] designed a bend-type piezoelectric micro-jet device to realize the active lubrication of space bearings and micro-fluid lubrication on demand. The minimum volume of oil ejected by the device could reach 0.012 L. Gross et al. [22] proposed a push-type piezoelectric micro-jet device, which used a nozzle with a diameter of 40 um and could control the droplet volume within the range of 100 PL to 250 PL. Cheng et al. [23] proposed a shear-type piezoelectric printhead with a special polarization design, and the maximum displacement was 400 nm at a driving voltage of 120 V_pp_.

Although the existing piezoelectric micro-jet devices have a series of advantages such as high precision of injection control and fast dynamic response speed, they are still prone to problems such as the existence of bubbles in the cavity, leading to the degradation of injection performance or even failure, and complicated disassembly after the failure of ceramics. In this work, a nozzle-driven piezoelectric micro-jet device is proposed, which adopts a drive mode of nozzle vibration with piezoelectric ceramic pasted at the nozzle part. Compared with the traditional cavity vibration method (in the traditional piezoelectric micro-jet, the vibrator is a part of the fixed surface of the cavity, and the nozzle does not move), the problem of reducing the injection performance due to the bubble entering the cavity is avoided. Moreover, the external nozzle has stronger interchangeability. When the ceramic is damaged, it is not necessary to disassemble the whole device and directly re-paste the new ceramic.

## 2. Structure and Mechanism

**Figure 1:**
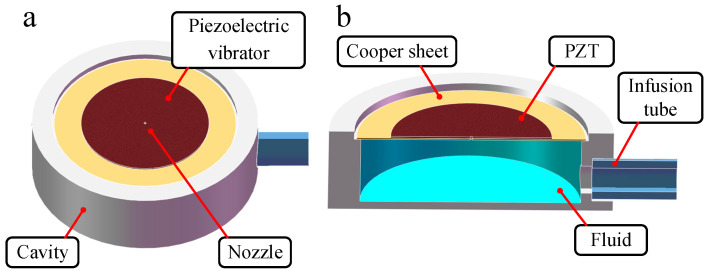
The overall structure and section view of piezoelectric jet device: (**a**) The overall structure of piezoelectric jet device; (**b**) the section view of piezoelectric jet device.

The nozzle-driven piezoelectric micro-jet device proposed in this work is shown in Figure 1, in which Figure 1a is the overall structure and Figure 1b is the section view of the device. The device is mainly composed of a piezoelectric vibrator, cavity and infusion tube, through which, viscosity lubrication oil is transported and stored in the cavity. The piezoelectric vibrator is the core component of the micro-jet device, which is composed of a PZT ceramic and a copper sheet and is fixed on the cavity via epoxy resin. In order to prevent the piezoelectric vibrator from short circuiting when it encounters viscous lubrication oil, the external surface of the PZT ceramic is insulated.

**Figure 2:**
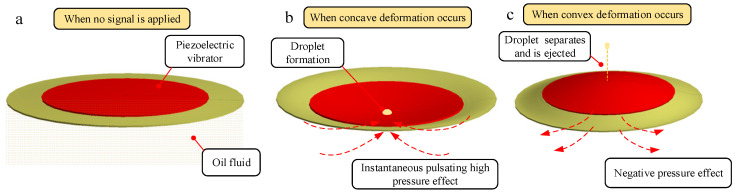
The ejection process of droplets: (**a**) When no signal is applied; (**b**) when concave deformation occurs; (**c**) when convex deformation occurs.

A nozzle is drilled in the center of the piezoelectric vibrator with a high-precision drilling machine, and the operation process of ejecting oil is shown in Figure 2. When the piezoelectric vibrator is not working, as shown in Figure 2a, the vibrator always keeps contact with the oil. When periodic square wave signals are applied in the vibrator, the vibrator vibrates and produces deformation, and the oil begins to periodically eject from the nozzle. As shown in Figure 2b, when the vibrator generates concave deformation, the oil is compressed, and instantaneous pulsating high pressure is generated in the cavity. Under the action of high pressure, the droplets are driven to converge at the nozzle and obtain kinetic energy. As shown in Figure 2c, when the vibrator generates convex deformation, the cavity expands, resulting in pulsating negative pressure in the cavity. Under the dual action of negative pressure and acquired kinetic energy, the liquid droplets are separated from the oil in the cavity and then fly out to form liquid droplets. According to the above analysis, the droplets are ejected periodically and uniformly with the periodic deformation of the piezoelectric vibrator.

**Figure 3:**
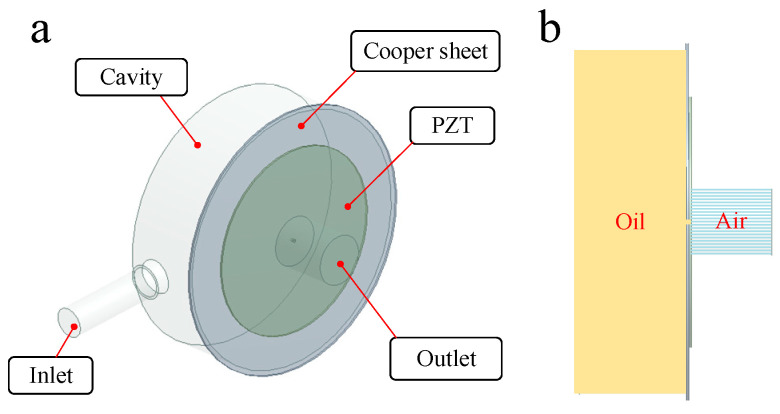
The axonometric and frontal view of the simulation model: (**a**) The axonometric view of the simulation model; (**b**) the frontal view of the simulation model.

In order to further reveal the principle of the piezoelectric micro-jet and study the influence of signal, oil viscosity and nozzle size on the injection performance, we use ANSYS software to carry out a two-phase, two-way fluid–structure coupling analysis on the designed piezoelectric micro-jet device. The simulation model is shown in Figure 3. As can be seen from the axonal diagram of the model in Figure 3a, the simulation model is composed of a cavity, copper sheet, PZT ceramics, liquid inlet and liquid outlet. The solid model of the cavity and infusion tube is removed, and the internal fluid model of the cavity and infusion tube is added. As can be seen from the front view of the model in Figure 3b, in order to simulate the injection of oil from the nozzle into the air, a fluid domain simulating the external air environment is added on the right side of the nozzle. The piezoelectric vibrator is set as the structural domain, and the cavity part is set as the fluid domain (oil). PZT-5H is selected as the material of PZT ceramics, and its Young′s modulus is 56 Gpa, Poisson’s ratio is 0.36 and density is 7600 kg/m^3^. The copper sheet is made of red copper with a Young′s modulus of 108 Gpa and Poisson′s ratio of 0.32. The thickness of copper and PZT ceramics is 0.2 mm. The transient structure analysis module and fluent module are used to simulate the two-phase, two-way fluid–structure coupling analysis.

In the transient analysis module, the fixed boundary condition is applied to the circumference of the copper sheet. The surface where the piezoelectric vibrator contacts the fluid domain is set as the fluid–solid interface, and a square wave pulse signal is applied to the piezoelectric vibrator. In the fluent module, the fluid domain is meshed via the sweep method, and the dynamic mesh method (remesh and smooth) is used to simulate the dynamic change in the fluid domain caused by the deformation of the piezoelectric vibrator through the coupling surface. The inlet of the fluid domain is set to the pressure inlet of 100 MPa constant pressure, and the outlet of the fluid domain is set to the pressure outlet of 0 MPa constant pressure.

**Figure 4:**
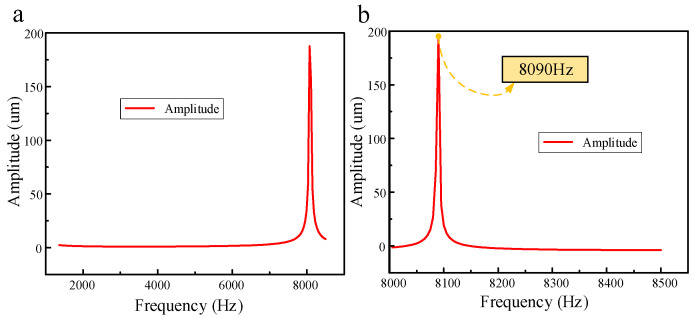
The results of the amplitude–frequency response: (**a**) The result of amplitude–frequency response in a large frequency analysis range; (**b**) the exact optimal frequency value.

In order to achieve the best injection effect, it is necessary to find an optimal injection frequency. The acoustic structure coupling analysis of the piezoelectric vibrator is carried out, that is, the bidirectional coupling frequency response analysis considering fluid reaction. First, the frequency with the largest amplitude is found within a large frequency range, and then, the frequency analysis range shrinks towards this frequency to obtain the exact optimal frequency value. The results of the amplitude–frequency response are shown in Figure 4. Figure 4a shows the result of the amplitude–frequency response in a large frequency analysis range, and Figure 4b shows the exact optimal frequency value. It can be seen that the vibration amplitude of the piezoelectric vibrator is maximum at 8090 Hz, so 8090 Hz is set as the optimal working frequency in this work.

**Figure 5:**
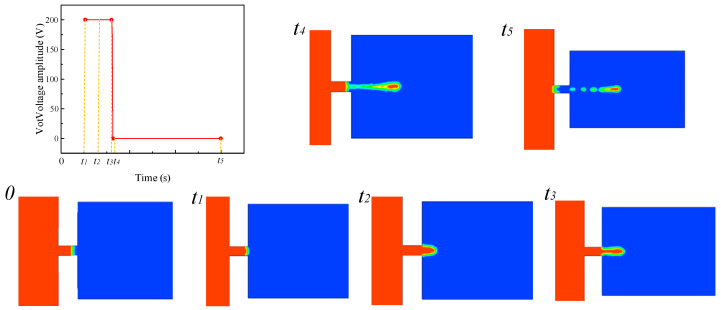
The single-droplet-injection two-phase flow diagram.

For the follow-up study on the influence of a single parameter on the injection state, the basic parameters are set as follows: voltage amplitude is 100 V, signal frequency is 8090 Hz, nozzle diameter is 0.4 mm, and oil viscosity is 200 Pa.s. The single-droplet-injection two-phase flow diagram of the piezoelectric micro-jet device under standard parameters is shown in Figure 5. The mechanism of the piezoelectric micro-jet will be elaborated upon in Figure 5. At *t* = 0 s, the device has no signal input and the liquid level remains stationary. At *t_1_*, the high-level pulse signal is applied to the piezoelectric vibrator, and the vibrator begins to produce concave deformation and squeezes the cavity to produce positive pressure, resulting in the oil beginning to converge to the nozzle. From *t_1_* to *t_3_*, the vibrator continues to receive positive voltage signal, the cavity keeps positive pressure and the oil continues to be ejected out. At *t_4_*, the pulse signal changes to a low level after the falling edge. At this time, the vibrator begins to produce convex deformation, and the cavity begins to expand, negative pressure begins to be generated and part of the oil begins to be sucked back. At *t_5_*, it can be seen that part of the droplets are sucked back, and the droplets are completely separated from the oil and ejected outward. The simulation results show the feasibility of the piezoelectric micro-jet and describe the generation process of droplets in detail.

**Figure 6:**
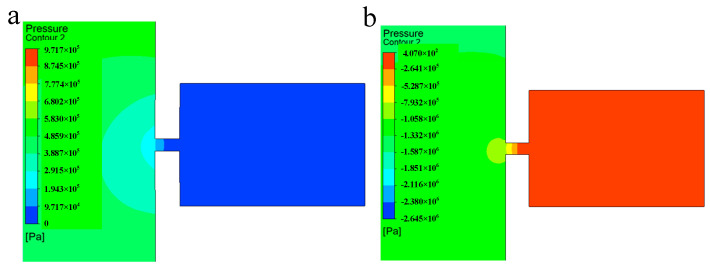
The single-injection pressure diagram: (**a**) The pressure diagram when the piezoelectric vibrator generates concave deformation; (**b**) the pressure diagram when the piezoelectric vibrator generates convex deformation.

The single-injection pressure diagram of the piezoelectric micro-jet device under standard parameters is shown in Figure 6, in which Figure 6a corresponds to the concave deformation of the piezoelectric vibrator, and Figure 6b corresponds to the convex deformation of the piezoelectric vibrator. As can be seen from Figure 6a, when the piezoelectric vibrator generates concave deformation, positive pressure is formed inside the cavity, which gives kinetic energy to the oil and squeezes the oil to the nozzle. As can be seen from Figure 6b, when the piezoelectric vibrator generates convex deformation, negative pressure is generated inside the cavity, which helps the droplet to separate from the oil. The simulation results are consistent with the previous analysis of droplet ejection.

**Figure 7:**
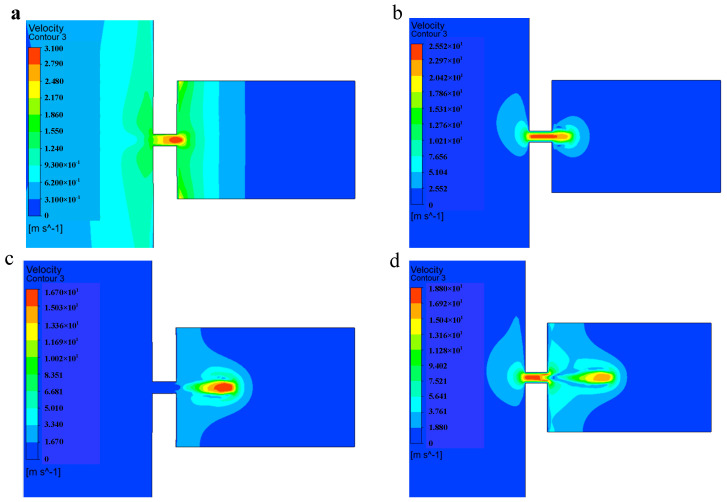
The injection velocity diagram: (**a**,**b**) The velocity diagram of the droplet during the convergence of the droplet at the nozzle; (**c**,**d**) velocity diagram of the droplet during the separation and ejection of the droplet from the oil.

The injection velocity diagram of the piezoelectric micro-jet device under standard parameters is shown in Figure 7. Figure 7a,b show the velocity diagram of the droplet during the convergence of the droplet at the nozzle. Figure 7c,d show the velocity diagram of the droplet during the separation and ejection of the droplet from the oil. From Figure 7a–d, it can be seen that with the gradual ejection of the droplet, the kinetic energy of the droplet is gradually lost due to the need to overcome the viscous resistance of the oil, and the velocity of the droplets is gradually reduced.

## 3. Performance Controlling

In the above section, we describe in detail the basic principle of the piezoelectric micro-jet and the formation process of single-droplet injection under standard parameters. In order to propose an injection performance control method for this device, this section will study the influence of various parameters—voltage amplitude, signal frequency, oil viscosity and nozzle diameter—on the injection performance. The injection performance considered in this work mainly includes droplet volume, droplet outlet velocity and ejection state. The outlet flow rate of the nozzle is integrated with time to obtain the droplet volume, and the droplet outlet velocity and ejection state are the droplet outlet velocity and ejection state at which the droplet is separated from the oil and is about to eject. In order to ensure that droplets can be fully ejected, the total duration of this simulation is set as 0.0003 s, which is about twice the period of a single pulse.

**Figure 8:**
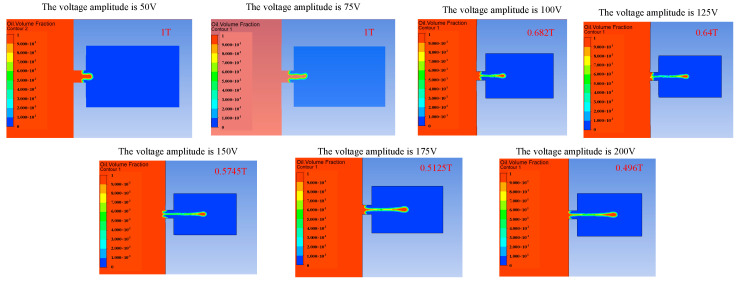
The change in droplet ejection state caused by the change in voltage amplitude.

**Figure 9:**
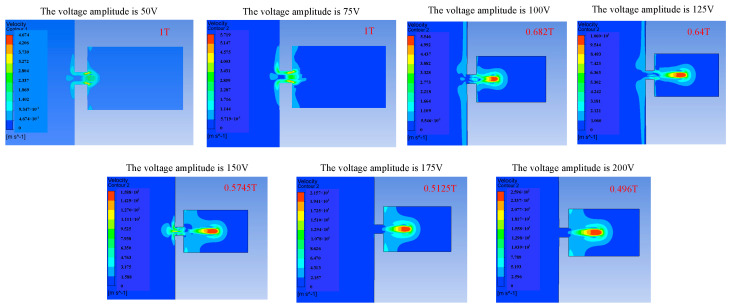
The change in droplet outlet velocity caused by the change in voltage amplitude.

**Figure 10:**
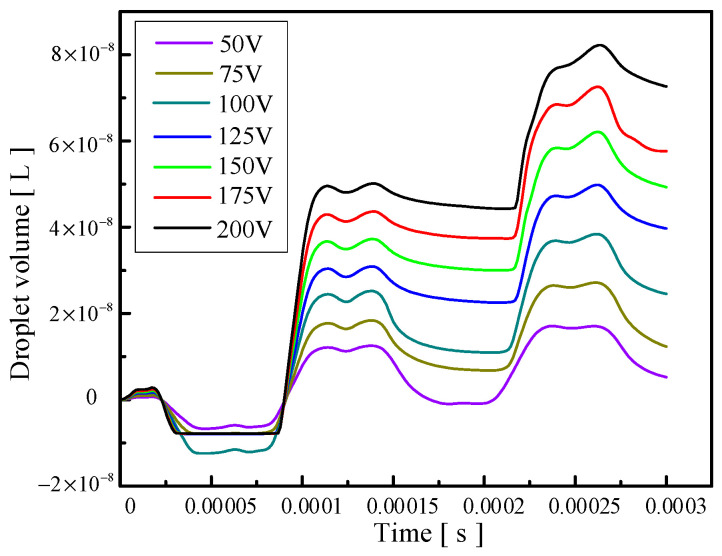
The change in the volume of droplets ejected caused by the change in voltage amplitude.

The voltage amplitude can affect the injection performance of the piezoelectric micro-jet device by changing the vibration amplitude of the piezoelectric vibrator. Firstly, the influence of voltage amplitude variation on the injection performance of the device is studied, and Figure 8, Figure 9 and Figure 10 reflect the influence of voltage amplitude variation on device injection performance. Figure 8 shows the change in the droplet ejection state caused by the change in voltage amplitude. It can be seen that when the voltage is too low, such as 50 V and 75 V, droplets cannot be formed and ejected within a total duration of 0.0003 s, while when the voltage amplitude is higher than 100 V, droplets can be ejected normally, and the larger the voltage amplitude, the earlier the droplet is ejected. If it is difficult to eject droplets, we can increase the voltage amplitude of the input signal. 

Figure 9 shows the change in droplet outlet velocity caused by the change in voltage amplitude. It can be observed that the droplet outlet velocity increases gradually with the increase in voltage amplitude. Figure 10 reflects the influence of voltage amplitude change on the volume of droplets ejected. It can be seen that the oil is first extruded inwards and then injected. With the increase in voltage amplitude, the volume of droplets ejected gradually increases, and the injection efficiency increases while the control accuracy of the droplets decreases. Therefore, if you want to reduce the difficulty of injection or improve the outlet velocity and injection efficiency of the droplets, you can appropriately increase the voltage amplitude, and if you want to improve the control accuracy of the droplets, you can appropriately reduce the voltage amplitude.

**Figure 11:**
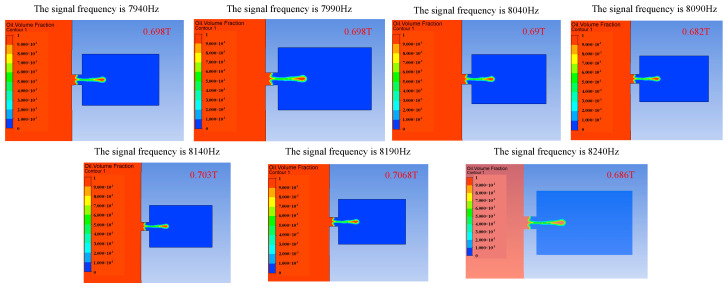
The change in droplet ejection state caused by the change in signal frequency.

**Figure 12:**
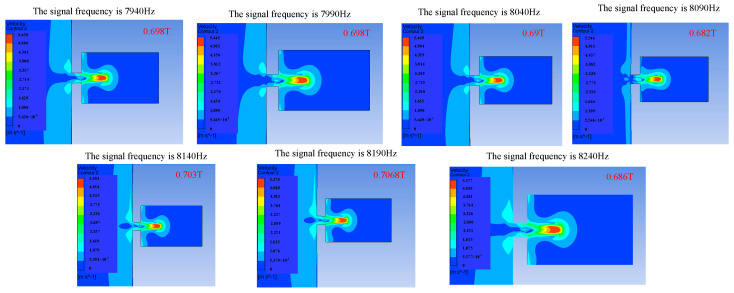
The change in droplet outlet velocity caused by the change in signal frequency.

**Figure 13:**
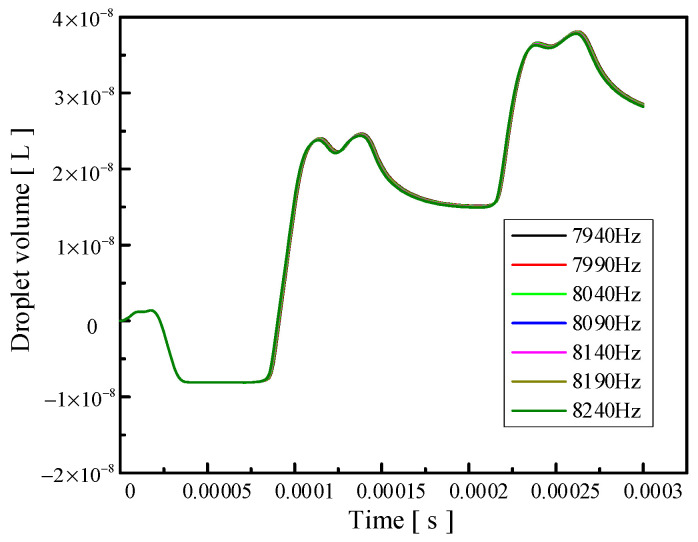
The change in the volume of droplets ejected caused by the change in signal frequency.

Signal frequency also has an influence on the injection performance. When the frequency of the applied signal is just under the frequency of the vibrator′s operating mode, the amplitude of the vibrator will increase, and the injection performance will be affected by changing the vibration amplitude of the vibrator. Figure 11, Figure 12 and Figure 13 show the influence of the change in signal frequency on the injection performance. It can be seen from the comprehensive analysis of Figure 11, Figure 12 and Figure 13 that the change in signal frequency has little influence on the injection performance of the droplet within the range of 7940–8240 Hz.

**Figure 14:**
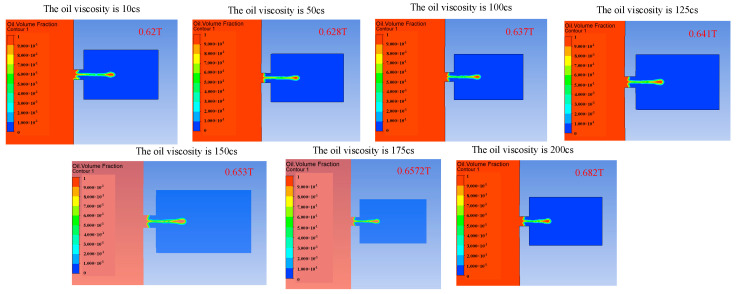
The change in droplet ejection state caused by the change in oil viscosity.

**Figure 15:**
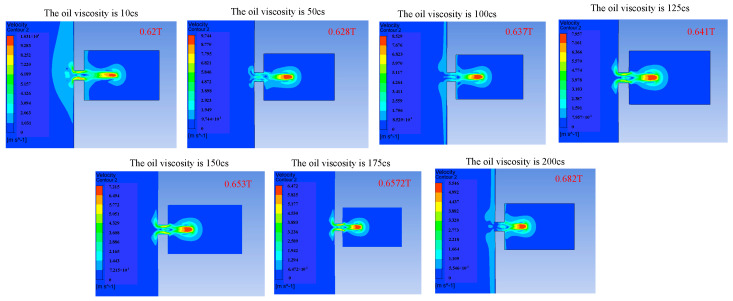
The change in droplet outlet velocity caused by the change in oil viscosity.

**Figure 16:**
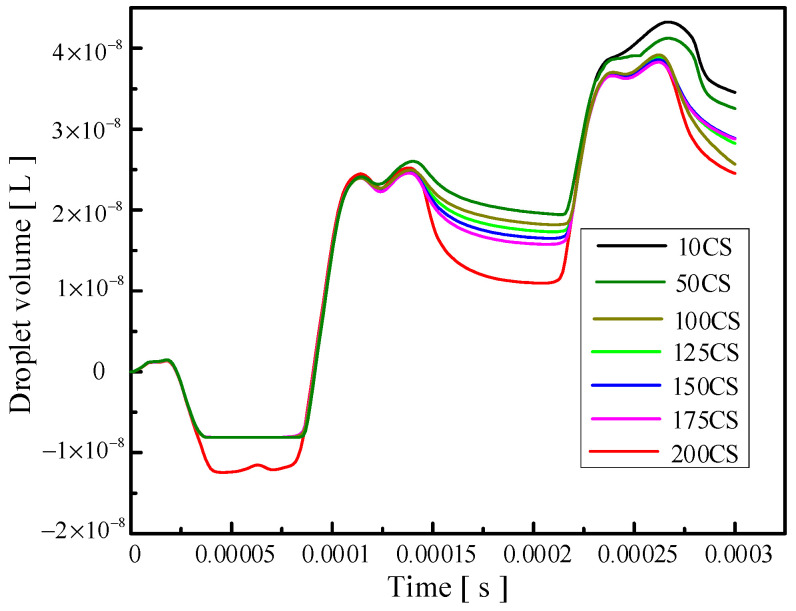
The change in the volume of droplets ejected caused by the change in oil viscosity.

Changing the viscosity of the oil will change the viscous resistance of the liquid, and a change in the viscous resistance will lead to a change in kinetic energy loss during the movement of the droplets. The influence of the change in the oil viscosity on the injection performance is shown in Figure 14, Figure 15 and Figure 16. Figure 14 reflects the influence of the change in oil viscosity on the ejection state of the droplets. It can be seen that with the increase in oil viscosity, the droplets are more difficult to be ejected, and the time of ejection is later. Figure 15 shows the influence of the change in oil viscosity on the outlet velocity of the droplet. With the increase in oil viscosity, the larger the kinetic energy loss of the droplet, the lower the outlet velocity. Figure 16 reflects the influence of the change in oil viscosity on the volume of droplets ejected. The analysis shows that when the viscosity is less than 100 Pa.s, the higher the oil viscosity, the smaller the volume of droplets ejected, and the higher the control accuracy but the lower the efficiency. When the oil viscosity is between 100 and 200 Pa.s, the oil viscosity has little influence on the volume of droplets. The comprehensive analysis shows that when the oil viscosity is greater than 100 Pa.s, the increase in the oil viscosity has a weak effect on the improvement in the control accuracy, but it will reduce the droplet outlet velocity and increase the difficulty of droplet injection, which will lead to the unsatisfactory injection performance of the device.

**Figure 17:**
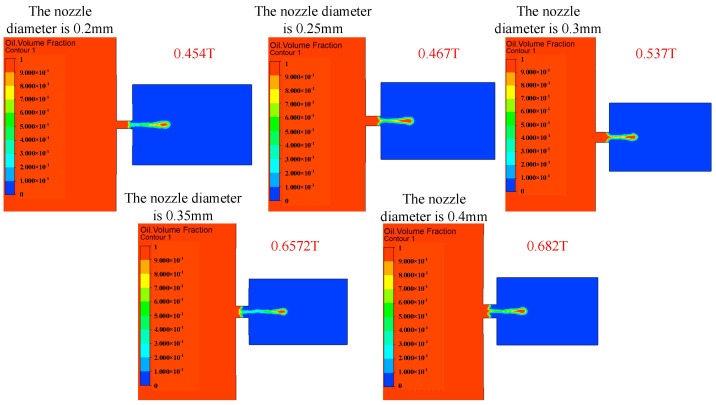
The change in droplet ejection state caused by the change in nozzle diameter.

**Figure 18:**
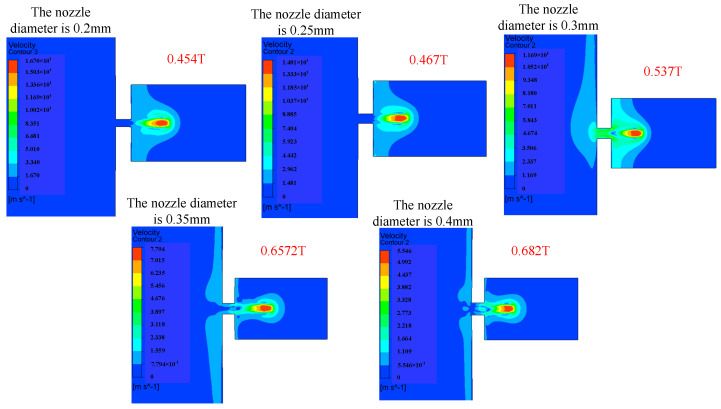
The change in droplet outlet velocity caused by the change in nozzle diameter.

**Figure 19:**
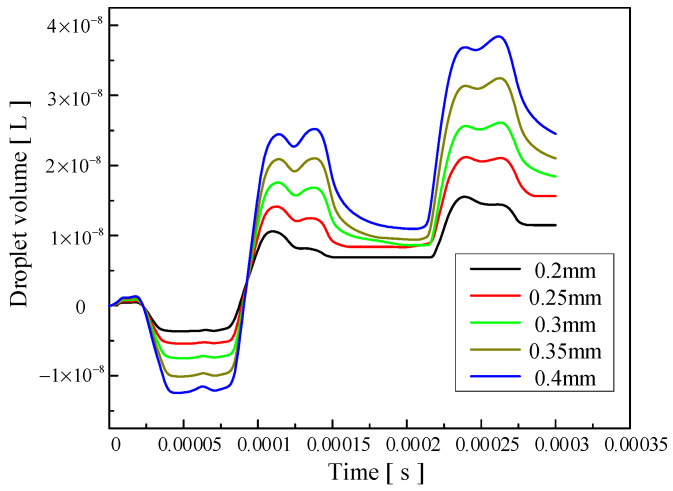
The change in the volume of droplets ejected caused by the change in nozzle diameter.

The influence of the change in nozzle diameter on the injection performance is shown in Figure 17, Figure 18 and Figure 19. Figure 17 shows the influence of the change in nozzle diameter on the ejection state of the droplet. With the increase in nozzle diameter, the stability of the droplet slightly decreases, and the tail begins to jitter. The larger the diameter of the nozzle, the greater the difficulty of the droplet ejection and the later the droplet ejection time. Figure 18 shows the influence of the nozzle diameter change on the droplet outlet velocity. With the increase in the nozzle diameter, the droplet outlet velocity gradually decreases. The nozzle diameter can be appropriately reduced if the initial kinetic energy of the droplet is to be increased. Figure 19 shows the influence of the nozzle diameter change on the volume of droplets. It can be seen that with the increase in the nozzle diameter, the volume of droplets ejected by the piezoelectric micro-jet device gradually increases. If the size of droplets ejected is to be increased to improve the injection efficiency, the nozzle diameter can be increased.

Based on the above simulation analysis, it can be seen that the frequency adjustment in a small range has little influence on the injection performance of the piezoelectric micro-jet device. In order to reduce the difficulty of injection, improve the injection efficiency and increase the droplet outlet velocity and volume, the following aspects can be considered: increasing the voltage amplitude of the input signal, reducing the nozzle diameter and using low viscosity oil. If the main consideration is to improve the accuracy of droplet control, we can try to reduce the voltage amplitude of the input signal, increase the nozzle diameter and use high-viscosity oil. The voltage amplitude and the nozzle diameter have significant influences on the droplet injection performance, while the oil viscosity has little influence on the droplet volume, namely the control accuracy, when it is greater than 100 Pa.s. However, as the viscosity of the oil increases, the difficulty of droplet injection increases and the outlet velocity decreases. Therefore, the piezoelectric micro-jet device is more suitable for ejecting droplets below 100 Pa.s. In the above simulation analysis, the micro-jet device can achieve the highest accuracy of 15.53 nL with a nozzle diameter of 0.2 mm. Under the voltage amplitude of 200 V, the maximum injection volume of 82.21 nL can be reached; that is, the device proposed in this work has both high efficiency and high precision.

## 4. Experiment Test

In the above section, the basic mechanism of micro-droplet injection is explained through ANSYS two-phase, two-way fluid–structure coupling analysis, and the influence of various parameters on the device′s injection performance is analyzed. In this section, an experimental system will be set up to shoot micro-droplet injection videos with industrial cameras to further verify the feasibility of the proposed device.

**Figure 20:**
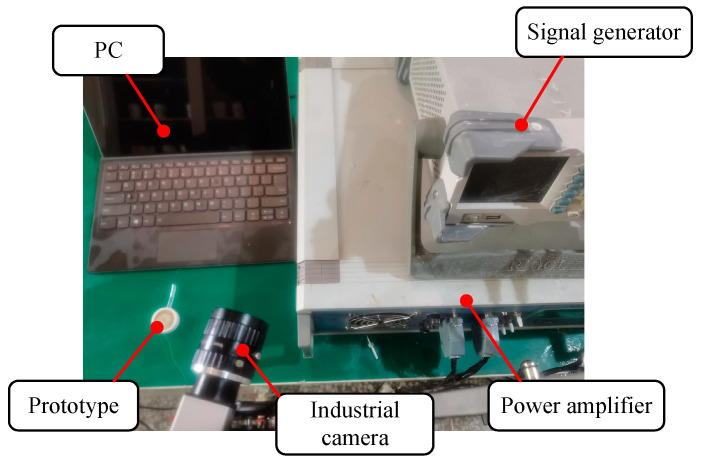
The experimental system.

In order to verify the feasibility of the proposed device, the experimental system is built, as shown in Figure 20. The experimental system mainly consists of a prototype, a personal computer (PC), a signal generator (DG4162, RIGOL, Beijing, China), a power amplifier (ATA-4051, Aigtek Inc., Xi’an, China) and an industrial camera. The experimental steps are as follows: firstly, the prototype is injected with a certain viscosity of viscosity lubrication oil; then, a given square wave signal is applied in the prototype through the signal generator and power amplifier, and then, the industrial camera is started to record the injection process. Finally, the video taken with the industrial camera is exported and analyzed using the PC.

**Figure 21:**
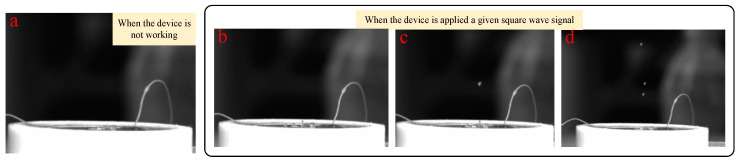
The results of the injection experiment: (**a**) The state of the piezoelectric micro-jet device when it is not working; (**b**) the droplet just being squeezed out of the nozzle; (**c**) the droplet separating from the oil and being ejected; (**d**) the formation of satellite droplets.

Corresponding to the above simulation analysis, the experimental parameters are set as follows: voltage amplitude, 100 V; square wave signal frequency, 728 Hz; nozzle diameter, 0.4 mm; fluid viscosity, 200 Pa.s. The results of the injection experiment are shown in Figure 21. Figure 21a shows the state of the piezoelectric micro-jet device when it is not working, and Figure 21b–d are the injection diagrams of the piezoelectric micro-jet device after the given square wave signal is applied. Figure 21b–d, respectively, correspond to the droplet just being squeezed out of the nozzle, the droplet separating from the oil and being ejected and forming satellite droplets, which are in accordance with the simulation in the above section, further verifying the correctness of the injection mechanism revealed. It can be seen from the observation that after the device works, the size of the droplets ejected is uniform and the injection state is stable, which can realize long-term continuous and stable injection, which confirms the correctness of the piezoelectric micro-jet principle and the feasibility of the proposed piezoelectric micro-jet device. The experimental video is attached as Video S1.

**Figure 22:**
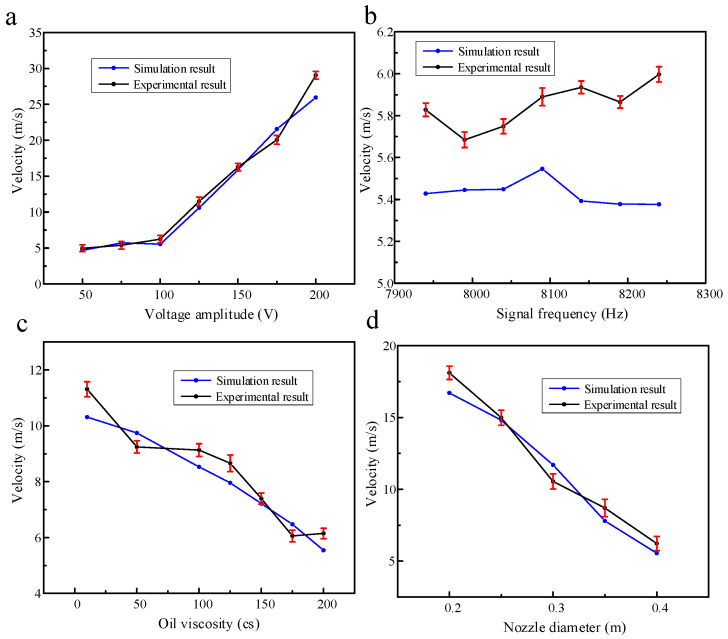
The experimental results of voltage amplitude, signal frequency, oil viscosity and nozzle diameter on droplet outlet velocity: (**a**) The experimental results of voltage amplitude on droplet outlet velocity; (**b**) the experimental results of signal frequency on droplet outlet velocity; (**c**) the experimental results of oil viscosity on droplet outlet velocity; (**d**) the experimental results of nozzle diameter on droplet outlet velocity.

**Figure 23:**
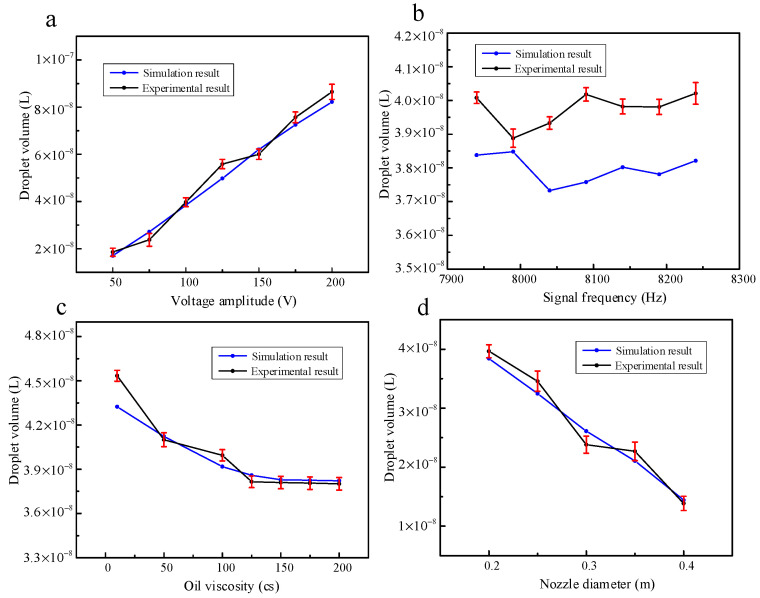
The experimental results of voltage amplitude, signal frequency, oil viscosity and nozzle diameter on droplet volume: (**a**) The experimental results of voltage amplitude on droplet volume; (**b**) the experimental results of signal frequency on droplet volume; (**c**) the experimental results of oil viscosity on droplet volume; (**d**) the experimental results of nozzle diameter on droplet volume.

In this work, the verification experiment is carried out according to the simulation content in the previous part. The main research is to investigate the outlet velocity and droplet volume of the piezoelectric micro-jet device under different voltage amplitudes, signal frequencies, oil viscosities and nozzle diameters. The standard experimental conditions are set as follows: voltage amplitude, 100 V; signal frequency, 8090 Hz; nozzle diameter, 0.4 mm; oil viscosity, 200 Pa.s. The experimental results of the effects of voltage amplitude, signal frequency, oil viscosity and nozzle diameter on droplet outlet velocity when other experimental conditions remain unchanged are shown in Figure 22. The experimental results are consistent with the simulation results in the above part, and the errors are all within the acceptable range. The maximum error of Figure 22a–d is 11.93%, 11.53%, 8.797% and 11.55%, respectively. The experimental results of the effects of voltage amplitude, signal frequency, oil viscosity and nozzle diameter on droplet volume when other experimental conditions remain unchanged are shown in Figure 23. The experimental results are consistent with the simulation results in the above section, and the errors are all within the acceptable range. The maximum error in Figure 23a–d is 12.17%, 6.92%, 4.86% and 9.61%, respectively.

The nozzle-driven piezoelectric micro-jet device proposed in this work is not like the traditional piezoelectric micro-jet device; due to the entry of bubbles, the stability of the device will gradually decline as the work progresses. Once the bubbles are entrapped, the air bubble grows via rectified diffusion and results in a malfunction [13]. In addition to excellent stability, the piezoelectric micro-jet device proposed in this paper also has good accuracy and ejection speed. The device has a maximum drop outlet velocity of 29.056 m/s at a voltage amplitude of 200 V and a maximum drop accuracy of 13.864 nL at a nozzle diameter of 0.2 mm. A piezoelectric micro-jet device with a pin joint designed by Deng et al. is a typical traditional piezoelectric micro-jet device. Under the condition of an operating frequency of 100 HZ and voltage amplitude of 100 V, 1 Pa.s adhesive is sprayed, and its maximum control accuracy can reach about 35 nL [24]. The piezoelectric micro-jet device proposed by Li et al. can obtain the injection speed of 4.7 m/s under the voltage amplitude of 130 V, and the control accuracy can reach 4.7 nL under the signal of a 50% duty cycle [25]. In summary, compared with the traditional piezoelectric micro-jet device, the nozzle-driven piezoelectric micro-jet device proposed in this work performs well in terms of control accuracy and ejection speed, while in terms of stability, the nozzle-driven piezoelectric micro-jet device is better because it is not affected by bubbles.

**Figure 24:**
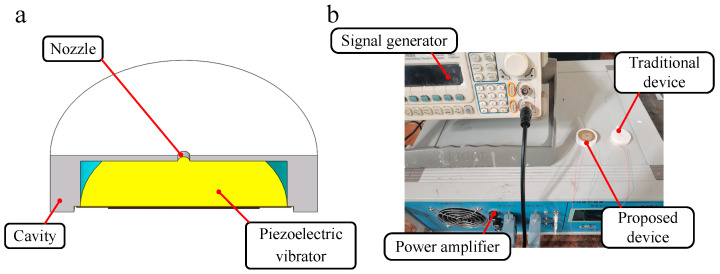
The internal structure of the traditional piezoelectric micro-jet device and the comparison experimental system: (**a**) The internal structure of the traditional piezoelectric micro-jet device; (**b**) the comparison experimental system.

**Figure 25:**
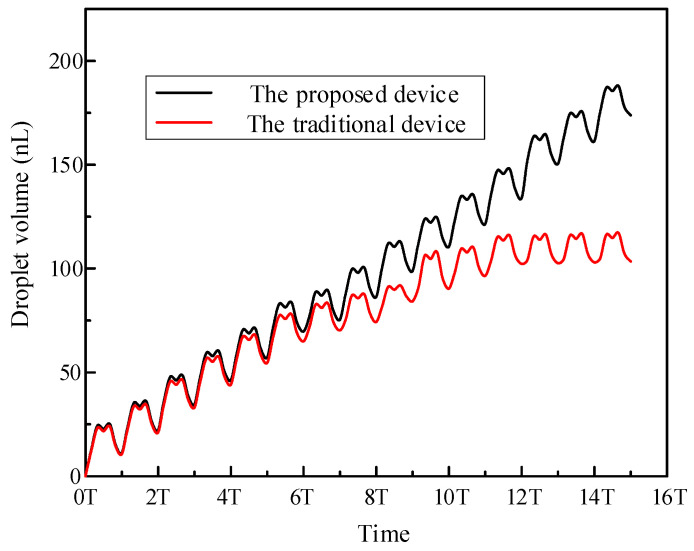
The experimental results of the comparison experiment.

In order to prove the stability of the proposed nozzle-driven piezoelectric micro-jet device, a traditional piezoelectric micro-jet device is fabricated in this work, and its internal structure is shown in Figure 24a. The cavity structure and experimental settings of the two sets of experimental devices are the same, and the experimental system built is shown in Figure 24b. The comparison experiment steps are as follows: under the settings of a voltage amplitude of 100 V, signal frequency of 8090 Hz, nozzle diameter of 0.4 mm and oil viscosity of 200 cs, the same continuous pulse signal is applied to the two sets of devices through a signal generator and power amplifier, and the volume of droplets ejected by the devices under multiple pulses is measured. The experimental results are shown in Figure 25. The analysis shows that both sets of devices have good ejection conditions in the initial few cycles, and both have high control accuracy. However, with the increase in the total working time, the ejection performance of the traditional piezoelectric micro-jet device gradually decreases due to the entry of bubbles until the device almost fails. Based on the above and experimental results, it can be seen that the nozzle-driven piezoelectric micro-jet device proposed in this work has good stability and control accuracy compared with the traditional piezoelectric micro-jet device.

## 5. Conclusions

In this work, a piezoelectric micro-jet device is proposed. Its unique nozzle driving mode makes the device have many outstanding advantages that traditional piezoelectric micro-jet devices do not have: (1) The external nozzle is different from the traditional cavity vibration type, which avoids the problem that the bubble enters the cavity and reduces the injection performance. (2) The external nozzle makes the structure of the device more compact, and the cavity cannot consider the assembly of the vibrator and the problem of an outlet wire. (3) The external nozzle has strong interchangeability, which can be replaced and repaired from time to time without remanufacturing and assembling the device.

Based on the brief description of the structure of the device and the process of micro-jet, ANSYS two-phase, two-way fluid–structure coupling analysis is carried out, and the mechanism of the piezoelectric micro-jet is elaborated upon in detail. On the basis of determining the optimal working frequency, the influence of voltage amplitude, input signal frequency, nozzle diameter and oil viscosity on the injection performance of the proposed device is studied, and a set of control methods is proposed based on this. A small range of frequency adjustment has little influence on the injection performance. Increasing the voltage amplitude, decreasing the nozzle diameter and decreasing the oil viscosity will reduce the difficulty of injection, increase the droplet outlet velocity and droplet volume and improve the injection efficiency of the device, but the control accuracy of the droplet will decrease. In addition, when the oil viscosity is greater than 100 Pa.s, it has little influence on the volume of droplets, namely, the control accuracy, while the influence on the difficulty of injection and the outlet velocity of droplets still exists. This work proved the correctness of the piezoelectric micro-jet mechanism and the feasibility of the proposed nozzle-driven piezoelectric micro-jet device through experiments and carried out the injection performance test experiment, which mainly investigated the effects of voltage amplitude, signal frequency, oil viscosity and nozzle diameter on the droplet outlet velocity and droplet volume. The experimental results showed that the piezoelectric micro-jet device has a maximum droplet outlet velocity of 29.056 m/s and a maximum droplet volume of 86.45 nL at a voltage amplitude of 200 V, while the device can achieve a maximum droplet accuracy of 13.864 nL at a nozzle diameter of 0.2 mm. From the above mentioned, it can be seen that the device combines high precision and high efficiency excellently. The experimental results coincide with the ANSYS simulation results, which confirms the correctness of the simulation experiment. Finally, a comparation experiment is carried out in this work, and the stability and superiority of the proposed device are verified by the experiment with the traditional piezoelectric micro-jet device.

## Data Availability

Data sharing is not applicable to this article.

## Supplementary Materials

The following supporting information can be downloaded at: https://www.mdpi.com/article/10.3390/mi14061267/s1, Video S1: experiment demo.
